# Can 3D diploid genome reconstruction from unphased Hi-C data be salvaged?

**DOI:** 10.1093/nargab/lqac038

**Published:** 2022-05-12

**Authors:** Mark R Segal

**Affiliations:** Department of Epidemiology and Biostatistics, University of California, 550 16th Street, San Francisco, CA 94143-0560, USA

## Abstract

The three-dimensional (3D) configuration of chromatin impacts numerous cellular processes. However, directly observing chromatin architecture at high resolution is challenging. Accordingly, *inferring* 3D structure utilizing chromatin conformation capture assays, notably Hi-C, has received considerable attention, with a multitude of *reconstruction* algorithms advanced. While these have enhanced appreciation of chromatin organization, most suffer from a serious shortcoming when faced with diploid genomes: inability to disambiguate contacts between corresponding loci on homologous chromosomes, making attendant reconstructions potentially meaningless. Three recent proposals offer a computational way forward at the expense of strong assumptions. Here, we show that making plausible assumptions about the components of homologous chromosome contacts provides a basis for rescuing conventional consensus-based, unphased reconstruction. This would be consequential since not only are assumptions needed for diploid reconstruction considerable, but the sophistication of select unphased algorithms affords substantive advantages with regard resolution and folding complexity. Rather than presuming that the requisite salvaging assumptions are met, we exploit a recent imaging technology, *in situ genome sequencing* (IGS), to comprehensively evaluate their reasonableness. We analogously use IGS to assess assumptions underpinning diploid reconstruction algorithms. Results convincingly demonstrate that, in all instances, assumptions are not met, making further algorithm development, potentially informed by IGS data, essential.

## INTRODUCTION

The three-dimensional (3D) architecture of chromatin within the eukaryotic nucleus is essential for numerous fundamental biological processes, including transcription, replication, development and even memory formation (1). Much of the current understanding of global principles of hierarchical chromatin organization derives from Hi-C and related assays (2–5). While many of these findings have emerged from analyses of the contact map – the matrix of pairwise interactions generated by a Hi-C experiment – there have a number of demonstrations of benefits in proceeding from a contact map to an *inferred 3D reconstruction*. In part, this added value derives from being able to superpose genomic attributes on the reconstruction. Examples include co-localization of genomic landmarks such as early replication origins in yeast (6,7), gene expression gradients in relation to telomeric distance and co-localization of virulence genes in the malaria parasite (8), the impact of spatial organization on double strand break repair (9), and elucidation of ‘3D hotspots’ corresponding to (say) overlaid ChIP-Seq transcription factor extremes which can reveal novel regulatory interactions (10).

Such potential has seen the development of a broad range of computational techniques for pursuing 3D genome reconstruction: a recent review (11) identified over 30 methods and there have numerous additions in subs equent years. These methods are broadly categorized into either *ensemble* or *consensus* approaches. The former generate a (large) collection of 3D solution structures that accord with the underlying Hi-C data, the intention being to capture the population of structures arising in a bulk cell experiment, with such experiments typically comprising 10^5^−10^6^ cells. However, as has been noted (12,13), whether such ensembles capture biological, including allelic (see below) variability, is unclear since decomposing variation into algorithmic and biological components is problematic.

**Figure 1.**
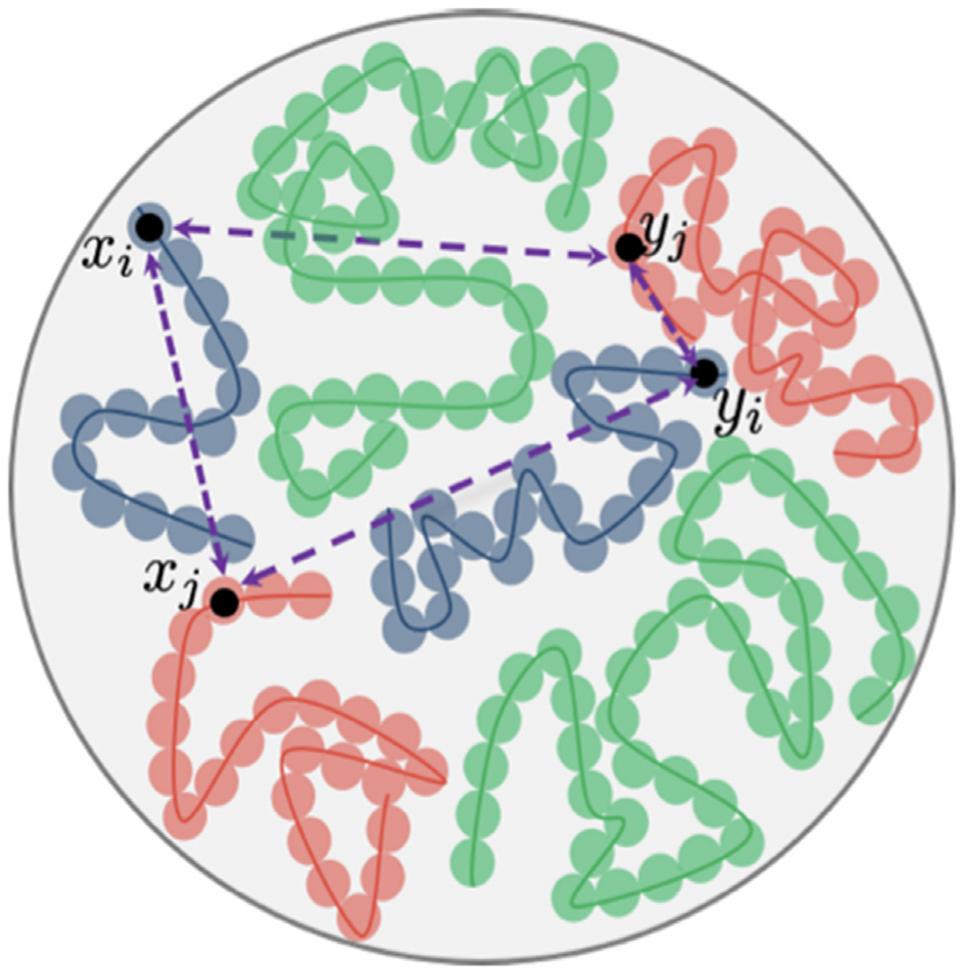
Three homologous pairs of chromosomes (red, green, and blue curves) are shown. For distances derived from unphased contact data the inferred distance between loci *i* and *j* corresponds to the sum of the four distances (depicted in purple) between the pairs of homologous loci (*x*_*i*_, *y*_*i*_) and (*x*_*j*_, *y*_*j*_). From (18). Copyright @Society for Industrial and Applied Mathematics. Reprinted with permission. All rights reserved.

Until recently, neither ensemble nor consensus reconstruction approaches have addressed the considerable challenge posed by typical Hi-C experiments conducted on *diploid* organisms. Since the Hi-C readout does not differentiate between allelic (homologous chromosome) copies, an observed contact between loci *i* and *j* corresponds to one of four possible events: either copy of locus *i* contacting either copy of locus *j*; see Figure 1. While accommodating this ambiguity seems essential to 3D reconstruction efforts, the fact of the matter is that the issue has been swept under the rug, either by being altogether ignored (implicitly imposing a haploid genome), or by assuming that chromosome homologs have a common 3D architecture (13–15).

The diploid reconstruction challenge has finally been met with the emergence of three pioneering computational approaches (16–18). In the Materials and Methods section, we briefly recapitulate these techniques, emphasizing the strong assumptions that are invoked to resolve allelic identifiability. We then show that making *a priori* reasonable assumptions about the components of homologous chromosome contacts provides the basis for salvaging conventional consensus-based, unphased reconstruction. In addition to averting issues associated with diploid 3D reconstruction algorithms, such rescue would have the added benefit of inheriting the sophistication of some existing unphased algorithms which, currently, afford substantive advantages with respect to resolution and capturing folding complexity (19). But while the needed assumptions are *prima facie* plausible, they are assumptions nonetheless. Accordingly, we exploit a newly developed imaging technology, *in situ genome sequencing* (IGS, (20), to comprehensively evaluate their reasonableness. This evaluation makes recourse to statistical approaches for comparing distance matrices and 3D configurations. We precede this assessment by also utilizing IGS imaging to appraise some of the assumptions underpinning the diploid-based reconstruction approaches. Findings from these programs are presented in the Results section, with concluding implications provided in the Discussion.

Our focus here is strictly on unphased Hi-C. While tools for phasing Hi-C data have recently been developed (16,21,22), thereby circumventing allelic ambiguity concerns, their use may be limited due to the sparsity of homolog-differentiating SNPs and the need for ultra-deep sequencing and attendant high resolution Hi-C maps required to effect phasing.

While it may seem misplaced to emphasize allelic identifiability issues in the face of the potentially more fundamental concern of inter-cell structural variation that is disregarded in consensus-based reconstruction (although (15) provide measures for evaluating adequacy of consensus solutions), the reasons for our focus are as follows. First, for 3D diploid reconstructions based on *single*-cell Hi-C (23,24) or SPRITE (25) assays the issue of inter-cellular structural variation becomes moot, but allelic ambiguity concerns persist. This concern is directly addressed by the work of (16) that we subsequently evaluate. While the sparsity of single cell proximity data has generally limited reconstruction applications to date, improved algorithms that accommodate zero inflation may help redress this (26). Second, existing methods for handling allelic ambiguity and effecting diploid reconstruction either pertain to distance-based consensus methods, or to single cell assays (where the distinction between consensus and ensemble is moot), and it is the reasonableness of their associated assumptions that we seek to assess. Appraising the impact of allelic ambiguity on the numerous ensemble based reconstruction methods is beyond the scope of this paper. Similarly, we do not attempt to address 3D diploid reconstruction for methods that operate in contact or neighborhood space without invoking distances.

## MATERIALS AND METHODS

We position description and assessment of emergent diploid 3D reconstruction approaches by initially briefly reviewing (implicitly haploid) consensus Hi-C based reconstruction methods below; in particular *multi-dimensional scaling* (MDS) based techniques. This is followed by subsequently detailing the assumptions required to computationally resolve allelic ambiguity and effect diploid reconstruction amongst existing approaches. The remainder of Materials and Methods then successively outlines the requirements needed to rescue haploid reconstruction methods, describes IGS data and processing, and showcases the statistical procedures applied to this data in order to evaluate the salvaging assumptions.

### 3D chromatin reconstructions from Hi-C data

We restrict attention to reconstruction of *individual* chromosomes; whole genome architecture can follow by appropriately positioning these solutions (27,28). As noted in the Introduction, in emphasizing consensus reconstructions from bulk cell experiments we disregard concerns surrounding inter-cellular structural variation.

The result of a Hi-C experiment is the *contact map*, a symmetric matrix $C = [C_{ij}]\in \mathbb {Z}_+^{n\times n}$ of contact counts between *n* (binned) genomic loci *i*, *j* on a genome-wide basis. Various approaches to contact matrix normalization have been proposed; the methods and issues dealt with here are agnostic to these. The 3D chromatin reconstruction problem is to use the contact matrix *C* to obtain a 3D point configuration $\mathbf {X} = \lbrace x_1, \ldots , x_n; x_i \in \mathbb {R}^3\rbrace$ corresponding to the spatial coordinates of loci 1, …, *n* respectively.

A common first step for many consensus based reconstruction approaches is conversion of the contact matrix into a *distance* matrix *D* = [*D*_*ij*_] (8,13,28–30), followed by solving the MDS (31) problem: position points (corresponding to genomic loci) in 3D so that the resultant interpoint distances ‘best conform’ to the distance matrix. A variety of methods have been used for transforming contacts to distances, with a number of approaches appealing to empirical observations and biophysical properties of DNA to invoke inverse power-law transfer functions: *D*_*ij*_ = (*C*_*ij*_)^−α^if*C*_*ij*_ > 0 (13,28,32). As has been emphasized (15), power-law relationships vary according to cell type, chromosome, organism, and resolution, making estimation of α important. However, while current diploid reconstruction methods both adopt such contact-distance conversion, they all prespecify values for the power-law index.

MDS operationalizes the notion of ‘best conforms’ via an objective function termed the *stress*, a standard version of which is:

(1) $$\begin{eqnarray*} \sigma (X) = \sigma (x_1,\ldots ,x_n) = {\sum _{i < j}} w_{ij} (\Vert x_i - x_j \Vert - D_{ij})^2 \end{eqnarray*}$$

where ‖ · ‖ denotes the Euclidean norm and $w$_*ij*_ are analogous to precision weights often taken as $D_{ij}^{-1}$ (15) or $D_{ij}^{-2}$ (13). More elaborate variants incorporate penalties to ensure loci with *C*_*ij*_ = 0 are not positioned too close (15). Several 3D reconstruction approaches use MDS as a building block (8,28–30), overlaying, for example, probabilistic (typically Poisson) modeling of contact counts. One such method, PASTIS (13), serves as a foundation for a diploid reconstruction approach.

### Computational approaches to diploid 3D reconstruction

As indicated, there have been three recently proposed computational approaches for pursuing diploid reconstruction from strictly unphased Hi-C data which we describe in turn with respect to attendant assumptions. While there are many facets to each of the methods, our focus is on evaluating the reasonableness of these assumptions that are key to enabling diploid reconstruction. The availability of even modest amounts of phased Hi-C data can appreciably alter the problem landscape, a topic we address further in the Discussion.

The first approach (16) assigns haplotypes based on the reasoning that unknown haplotypes can be imputed from ‘neighboring’ (in terms of genomic distances) contacts by assuming that the two homologs would *typically* contact different chromosome partners. We term this the DCP assumption. The underpinnings of this assumption are unclear. While it would follow from a random model of chromosome arrangement, this is contrary to interphase nuclear organization being evolutionarily conserved and strongly influenced by gene density and chromosome size in mouse and human (33), organisms relevant to our subsequent IGS analyses. Indeed, such organizing principles would tend to result in comparably sized and gene dense homologs occupying proximal territories. We provide empiric assessment of the DCP assumption in the Results.

The second approach (17) builds on the abovementioned PASTIS method by applying two constraints to the underlying Poisson log-likelihood. The first constraint attempts to impose chromatin connectivity by minimizing the variance in the distance between locus positions that correspond to neighboring genomic loci. While similar constraints have previously been employed (34), capturing contiguity by imposing that the 3D solution lie on 1D curve, achieved using principal curve metric scaling (19), arguably affords a more principled and flexible approach.

The second constraint is central to disambiguating diploid genome contact counts and is based on the tendency of homologs of most organisms to reside in distinct chromosome territories (33,35). In something of a leap, this observation is used to contend that the separation between chromosome centers of mass is *expected* to be similar to the corresponding separation between homologs. We term this the ECM (equal centers of mass) assumption which, again, can be tested using IGS data. This constraint, which can be interpreted as a log-prior in a Bayesian context where the distance between homolog centers of mass for chromosome *C* is normally distributed with mean *r*_*C*_, is formulated as

(2) $$\begin{eqnarray*} h_C(\mathbf {X}) = \max \Big \lbrace 0, \Big ( r_C - \big \Vert \bar{X}_M - \bar{X}_P \big \Vert \Big ) \Big \rbrace \end{eqnarray*}$$

(3) $$\begin{eqnarray*} h(\mathbf {X}) = \sum _{C \in \cal {K}}h_C (\mathbf {X})^2 \end{eqnarray*}$$

where, in (2), ‖ · ‖ is the Euclidean norm, |*C*_*M*_| gives the number of points in the 3D reconstructed maternal homolog $\mathbf {X}_i, \ i \in C_M$, with $\bar{X}_M = {\frac{1}{|C_M|}} \sum _{i \in C_M} \mathbf {X}_i$ its center of mass, and analogous definitions for the paternal homolog, while $\cal {K}$ in (3) denotes the set of autosomes.

The parameter *r*_*C*_ in (2) is crucial for PASTIS diploid reconstruction. In the setting of interest here, where only unphased Hi-C data is available, (17) set this predefined scalar as the mean distance between chromosome centers of mass of a 3D reconstruction that ignores ploidy, invoking the assumption that this distance is similar to that between homologs. We assess this presumed similarity, the ECM assumption, using IGS data in the Results section.

The third approach (18) provides a comprehensive mathematical treatment of identifiability concerns that derive from phase ambiguity, coupled with constraints and data augmentations needed to effect 3D diploid reconstruction. Here such reconstruction is effected using embedding (eigen decomposition) techniques applied to the diploid (2*n* × 2*n*) Gram matrix constructed from the observed *n* × *n* contact matrix and the constraints. Analogous embedding methods having previously been used in the (implicitly) haploid setting (15). In addition to a constraint pertaining to 3D distances between neighboring genomic loci, identifiability is achieved via a constraint derived from *multi-way* contact assays such as SPRITE (36) or GAM (37).

**Figure 2.**
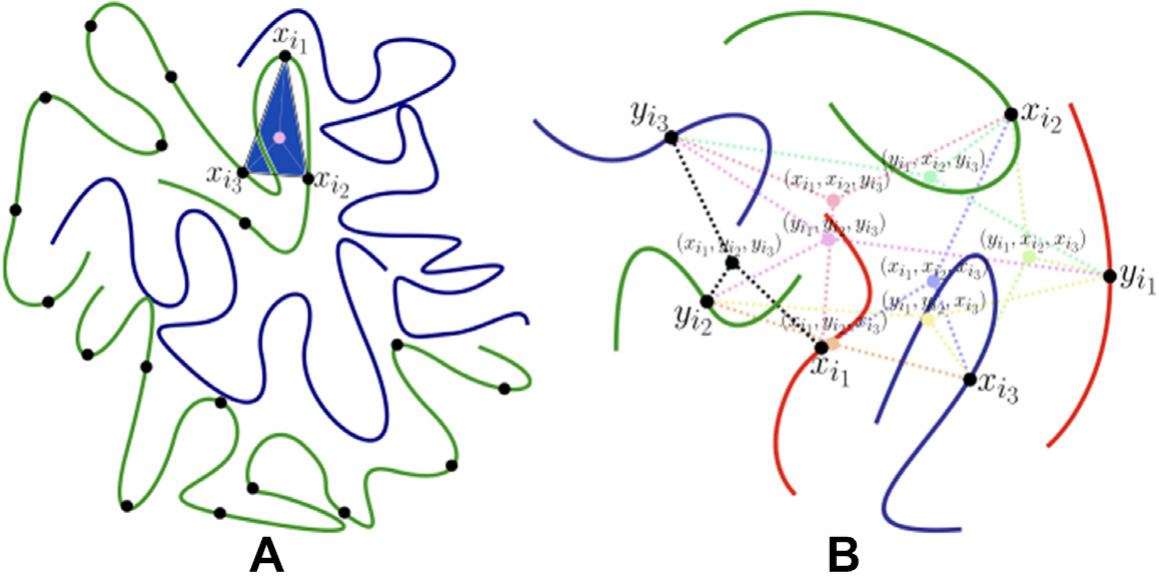
(**A**) Phased setting. Two chromosomes (green, blue curves) with three loci $x_{i_1} , x_{i_2} , x_{i_3}$ on the same chromosome are shown. In the phased setting, the higher-order distance $D_{x_{i_1} x_{i_2} x_{i_3}}$ is defined as the sum of the distances (pink dashed lines) of the three loci to their centroid (pink circle). (**B**) Unphased setting. Three chromosomes (green, blue and red curves) with three *homologous* loci $(x_{i_1}, y_{i_1}), (x_{i_2}, y_{i_2}), (x_{i_3}, y_{i_3})$ depicted. These give rise to eight possible higher-order distances, based on the eight centroids, illustrated by the colored dashed lines. The higher-order distance $D_{i_1 i_2 i_3}$ is defined as the minimum of these eight distances, here achieved by the three black dashed line segments. From (18). Copyright @Society for Industrial and Applied Mathematics. Reprinted with permission. All rights reserved.

To use multi-way contacts for (distance based) reconstruction, a corresponding multi-way distance is needed. For a three-way (intra-chromosomal) interaction in the phased setting (18) operationalize the higher-order distance between 3D loci as the sum of their distances to their centroid; see Figure 2A. We comment on alternate formulations using point-set diameters or tensor distance in the Discussion. In the unphased setting there are eight possible higher-order distances resulting from the 8 centroids pertaining to the three pairs of homologous loci; see Figure 2B. Of these eight, the smallest is chosen as the defining distance, based on an assumption that one of the three-way interactions (*triplets*) constitutes the majority of the observed contact frequency count. We term this the Dom8 assumption, and use IGS data for evaluation thereof.

### Rescuing unphased reconstruction methods

For phased Hi-C data the allele-aware contact matrix for a homolog pair with *n* bins is

(4) $$\begin{eqnarray*} C = \left[\begin{array}{c|c} C_{MM} & C_{MP} \\ \hline C_{PM} & C_{PP} \end{array} \right] \end{eqnarray*}$$

where *C* is 2*n* × 2*n* and each intra- or inter- maternal (M) or paternal (P) homolog block is *n* × *n*. Let *D* designate the correspondingly partitioned distance matrix obtained, for example, by power-law transformation of *C*. As noted by (18), for unphased diploid data a naive approach is to assume that the four distances that make up the observed composite distance *D*_*ij*_ are equal. While earlier single-cell imaging studies make such an assumption untenable, here we posit alternate, *a priori* plausible assumptions about the composite contacts and attendant distances, and then turn to more recent imaging data for validity checking.

From Figure 1 the composite distance due to allelic ambiguity is the sum *D*_*ij*_ = ||*x*_*i*_ − *x*_*j*_||^2^ + ||*x*_*i*_ − *y*_*j*_||^2^ + ||*y*_*i*_ − *x*_*j*_||^2^ + ||*y*_*i*_ − *y*_*j*_||^2^ which we rewrite as $D_{ij} = D_{M_i M_j} + D_{M_i P_j} + D_{P_i M_j} + D_{P_i P_j}$. (While Figure 1 depicts the general scenario where loci *i*, *j* reside on different chromosomes, all considerations specialize to the setting where they reside on the same chromosome.) Due to the abovementioned tendency for chromosomes to occupy distinct, spatially separated territories within the nucleus we assume that the intra-homolog component distances are appreciably smaller than their inter-homolog counterparts. Further, based solely on underlying sequence similarity and disregarding consequential considerations such as epigenetic and local nuclear environmental factors, we assume that the maternal and paternal alleles have similar configurations. Thus, our salvaging distance assumptions are

(5) $$\begin{eqnarray*} D_{M_i M_j} \ll D_{M_i P_j} \qquad D_{P_i P_j} \ll D_{P_i M_j} \end{eqnarray*}$$

(6) $$\begin{eqnarray*} D_{M_i M_j} \approx D_{P_i P_j} \end{eqnarray*}$$

Mapping (5-6) back to the underlying contacts gives $C_{M_i P_j} / C_{M_i M_j} \approx C_{P_i M_j} / C_{P_i P_j} \approx 0$ and $C_{M_i M_j} \approx C_{P_i P_j}$. Thus, our *observed* *n* × *n* contact matrix *C*_*n*_ will approximate *C*_*MM*_ or *C*_*PP*_ and performing 3D reconstruction thereon will recapitulate the (assumed) common architecture.

Note that these assumptions are framed in terms of *approximations*. Recall that our objective was salvaging existing 3D reconstruction approaches that utilize unphased Hi-C data. Such reconstructions, as well as their analogs based on phased Hi-C data, are inherently approximate, being subject to numerous sources of uncertainty impacting both data (biological, technical variation) and methodology (algorithm choice, tuning) components. However, formal analysis of what constitutes adequate degrees of agreement is problematic in view of an absence of *linked* gold standards: while we use IGS, described next, as a proxy we would require Hi-C data on the same samples to effect such quantification. So, in appraising assumptions we are limited to statistical tests on IGS 3D configurations and attendant distance matrices.

### In situ genome sequence imaging

**Figure 3.**
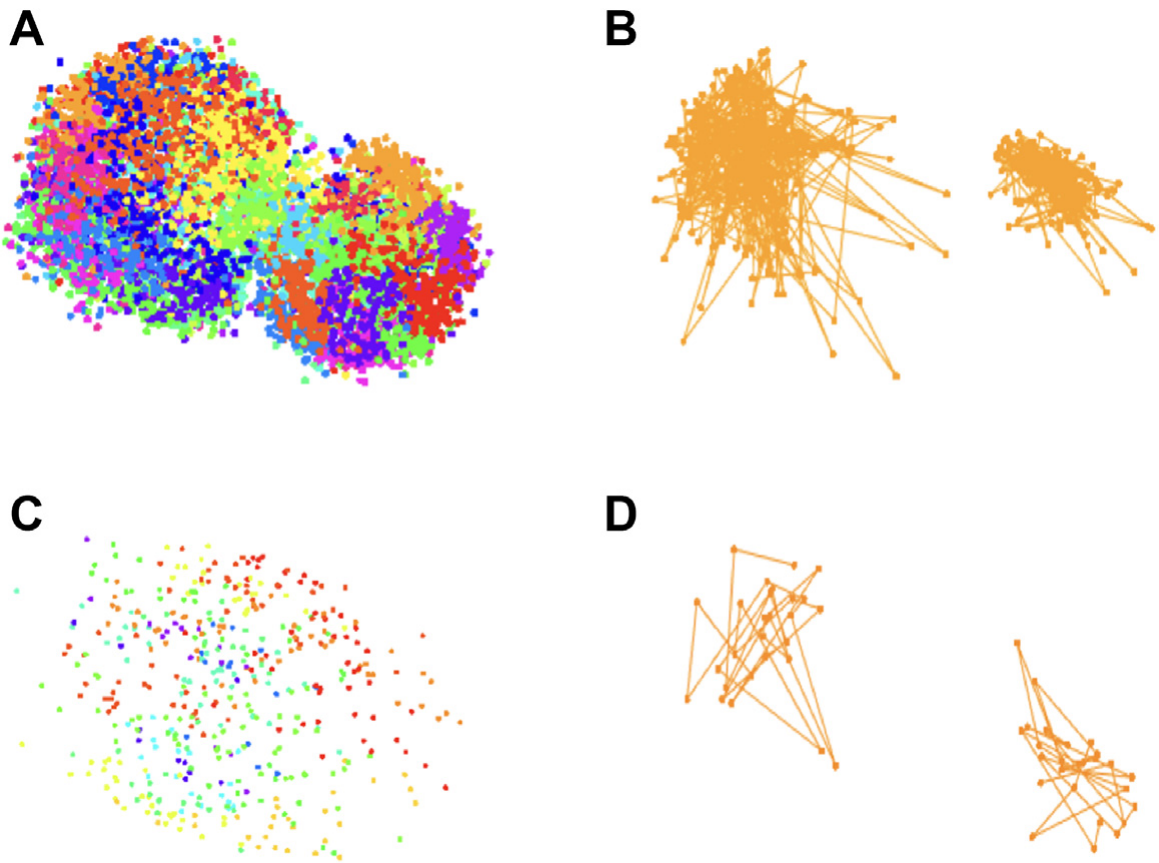
IGS 3D coordinates. (**A**) Mouse zygote 8 with 7284 phased reads color coded for chromosomes 1 through 19. (**B**) Zoomed out view for chromosome 3 maternal and paternal homologs (560 total reads). (**C**) PGP1f cell 85 with 461 phased reads color coded for chromosomes 1 through 22. D: Zoomed out view for chromosome 3 maternal and paternal homologs (53 total reads).

To evaluate the distance-based assumptions we turn to IGS (20) which, crucially, provides whole genome, high resolution, allele specific spatial maps and offers advantages over other recent approaches (38,39). Data consisting of 3D coordinates, and corresponding genomic coordinates, for the two systems analyzed—106 human fibroblasts (PGP1f cells) and 24 mouse zygotes—were obtained from https://www.science.org/doi/10.1126/science.aay3446 (Supplementary Tables S1 and S2, last accessed 5 April 2022). Figure 3 displays whole genome as well as an illustrative homolog pair (chromosome 3) image data for the zygote and cell with the greatest number of reads for mouse and human respectively. In using the term ‘reads’ we are adhering to the above data sources and (20), to which details of the IGS assay are deferred.

From the standpoint of evaluating our salvaging distance assumptions above some salient considerations emerge from Figure 3. As is evident from panel A, the maternal and paternal pronuclei have yet to fully fuse. This will greatly distort the comparison of intra- and inter- homolog distances, needed to assess (5). So, in order to utilize the relatively rich zygote data, we artificially impose fusion by translating one pronucleus to the other so that they share a common center of mass. We note that while apparent scale differences between the pronuclei are accommodated by some of our statistical testing procedures, rescaling so that the pronuclei have a common scale was also performed so as to permit the direct distance-based comparisons needed, for example, to assess the DCP assumption.

This artificial fusion procedure is admittedly crude and may not reflect the complexity of chromatin reorganization following fertilization (40). So, it may seem preferable to focus exclusively on PGP1f cells. However, as is apparent from Figure 3, their read counts are appreciably sparser than those for mouse zygotes, respective medians being 257 and 3909. This relative sparsity is exacerbated by our distance assumption evaluation requiring alignment in terms of genomic coordinates: *M*_*i*_ and *P*_*i*_ (similarly *M*_*j*_ and *P*_*j*_) in (5-6) need to correspond to the same genomic locus. To achieve this binning, akin to that employed in constructing Hi-C contact matrices, must be deployed. Given that IGS imaging does not preferentially target common loci on companion homologs, restricting to bins *shared* between homologs results in sparse data for all PGPf1 cells, when undertaken at 1 Mb resolution. We therefore use both systems in a manner that reflects their respective strengths for assumption evaluation.

### Formal comparisons of distance matrices and 3D structures

We use two main approaches for testing our proposed salvaging of 3D genome reconstruction from unphased Hi-C data, drawing on phased 3D chromosome configurations obtained from IGS. The rescue effort is framed in terms of equality of maternal and paternal distance matrices (6), which we construct using binned IGS data as above, and test using the Generalized RV test (GRV, (41)), briefly described below. Additionally, we graphically examine inter- versus intra- homolog distances to appraise the territory-driven assumptions (5).

Irrespective of the outcome of this GRV testing program it is purposeful to directly compare the observed maternal and paternal 3D IGS structures, as opposed to putative 3D reconstructions from distance matrices. This is on account of GRV test results being impacted by operating characteristics of the test, here notably sparsity and to eliminate the impact of the reconstruction process itself. We effect such comparison, after binning IGS 3D coordinates to provide common (between allele) genomic loci, using Procrustes alignment and attendant permutation testing, further described subsequently.

#### Generalized RV test

The GRV test for equality of distance matrices can handle a variety of data types and distance measures and offers improved power over competing tests in many settings (41). Let *G*_*M*_, *G*_*P*_ be the *n* × 3 matrices with rows the 3D coordinates for the *n* (common, binned) genomic loci obtained from IGS imaging. The precursor RV statistic is developed as a matrix extension of Pearson’s correlation:

(7) $$\begin{eqnarray*} \phi _R(G_M,G_P) &=& {\rm RV}(G_M,G_P) \nonumber \\ &=& \frac{{\rm tr}\big (G_M^T G_P G_P^T G_M \big )}{ ||G_M^T G_M||_F ||G_P^T G_P||_F} \nonumber \\ &=& \frac{{\rm tr}\big (G_M G_M^T G_PG_P^T \big )}{ ||G_M G_M^T||_F ||G_P^TG_P^T||_F} \end{eqnarray*}$$

Since $G_MG_M^T = - 1/2 A D_{MM}^2 A$ where *A* = (*I*_*n*_ − *J*_*n*_/*n*) with *I*_*n*_ the *n* × *n* identity matrix and *J*_*n*_ the *n* × *n* matrix of ones, and similarly for $G_PG_P^T$, ϕ_*R*_ is completely determined by the intra-homolog distance matrices *D*_*MM*_, *D*_*PP*_. The generalized RV test simply replaces the underlying Euclidean distances with any distance measure although for our spatial applications we do not consider non-Euclidean distances. An important feature of the GRV test is that inference can utilize closed-form *p*-value approximations. These are derived by matching the first three moments of the exact null distribution obtained from all *n*! distance matrix row (or column) permutations to a Pearson type III distribution which captures appropriate skewness characteristics, and are readily computed via attendant analytical results.

#### Procrustes distance testing

There are many sources describing Procrustes analysis (e.g. (31)) which facilitates assessing correspondences between shapes. In comparing 3D chromosome configurations we are interested in *reflection similarity shape*, under which two configurations that only differ by a reflection, rotation, translation and scaling are deemed equivalent. The closeness of *G*_*M*_ and *G*_*P*_ can be measured by how far apart corresponding points are, after optimizing for the allowed transformations. Initially, ignoring scaling, this gives rise to the criterion:

(8) $$\begin{equation*} \min _{\mu ,Z} ||G_P - (G_M Z + 1\mu ^T)||_F \end{equation*}$$

where *Z* is a 3 × 3 orthogonal matrix and μ is a 3-vector of translation coordinates. Closeness is measured by the Frobenius norm: $||X||^2_F = {\rm trace} (X^TX) = \sum _{ij} x^2_{ij}$. Let $\bar{g}_M, \bar{g}_P$ be the respective column means of *G*_*M*_, *G*_*P*_ and $\tilde{G}_M,\tilde{G}_P$ centered versions obtained by column mean subtraction. Let the singular value decomposition of $(\tilde{G}_M)^T \tilde{G}_P = U{\Lambda }V^T$. Then the solution to (8) is

(9) $$\begin{eqnarray*} \hat{Z} = UV^T \end{eqnarray*}$$

(10) $$\begin{eqnarray*} \hat{\mu } = \bar{g}_P - \hat{Z} \bar{g}_M \end{eqnarray*}$$

Based on the form of the solution (9-10) we can work with $\tilde{G}_M,\tilde{G}_P$ and disregard location. Then, after re-introducing scaling, we arrive at our Procrustes distance equality criterion:

(11) $$\begin{equation*} \phi _P(\tilde{G}_M,\tilde{G}_P) = \min _{\beta ,Z} ||\tilde{G}_P - \beta \tilde{G}_M Z||_F \end{equation*}$$

with solutions $\hat{Z}$ as in (9) and $\hat{\beta }= {\rm trace}({\Lambda }) / ||\tilde{G}_M||^2_F$. Inference for $\phi _P(\tilde{G}_M,\tilde{G}_P)$ makes recourse to permutation which we effect using the protest function of the R package vegan (42).

## RESULTS

We use IGS data to assess the respective assumptions underpinning the three 3D diploid reconstruction approaches, as well as those facilitating salvage of haploid 3D reconstruction approaches outlined above. In each instance we find that the assumptions are generally *not* supported.

### Current diploid reconstruction technique assumption evaluation

#### Tan et al.

We evaluated the DCP assumption using both fused, rescaled mouse zygotes and human fibroblast PGP1f cells by simply determining whether or not the closest chromosome to a given homolog was its partner. Here closest is measured via Euclidean distance between chromosome centroids (centers of mass).

Eleven of the 24 mouse zygotes possessed chromosomes for which the closest neigboring chromosome amongst the 37 (=(2*19) − 1) competing chromosomes was its homolog partner. This greatly exceeds expectation under a random chromosome positioning assumption according to an exact one-sample binomial test, even after stringent adjustment for multiple testing (over chromosomes) with a Bonferroni corrected *P*-value <2.32 × 10^−13^.

Similarly, for 22 of the 106 human PGP1f cells the closest neighboring chromosome amongst the 43 (=(2*22) − 1) competing chromosomes was its homolog partner. Again this greatly exceeds expectation under a random chromosome positioning assumption, tested as above, with Bonferroni corrected p-value <3.75 × 10^−10^. However, this result is subject to the uncertainty accompanying the sparsity of PGP1f data.

Hence, the DCP assumption that homologs are closer to chromosomes other than their homolog partner, is not supported. As previously indicted, this is not surprising in view of chromosomal nuclear positioning being strongly influenced by gene density and size, properties clearly shared by homolog pairs.

#### Cauer et al.

The ECM assumption is crucial to the approach of (17) for effecting diploid 3D reconstruction. This assumption, which asserts that distance between non-homologous chromosome centroids is similar to the distance between homolog centroids is also amenable to testing based on IGS imaging. We effect such testing by simply computing the respective distances (per mouse zygote or PGP1f cell) for the two groups (homolog, non-homologs) and comparing these using two-sample t-tests.

**Figure 4.**
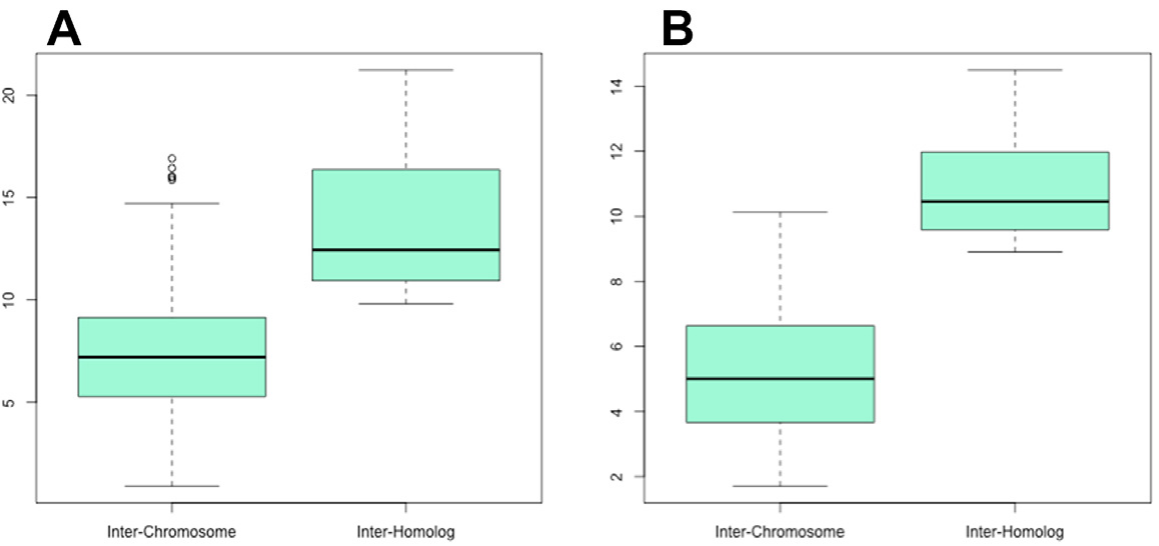
Distances (Y axis) between chromosome centroids for non-homologous and homologous pairs: (**A**) mouse zygote 24; (**B**) PGP1f cell 59.

Overall, 92% (=22/24) zygotes exhibit statistically significant (*P* < 0.05) distance non-overlap whereby inter-homolog centroid distances exceed inter-chromosome centroid distances, with 46% (=11/24) of these withstanding Bonferroni multiple testing correction (*P* < 0.05/24). A boxplot showcasing these differences for the most extreme instance is shown in Figure 4 A.

In assessing ECM using PGP1f cells we again confront sparsity issues. Erring on the side of inclusivity, we only exclude chromosomes with <3 reads thereby retaining 86 of 106 cells. Of these, 69% (=59/86) cells exhibit statistically significant distance non-overlap with 21% (=18/86) withstanding Bonferroni correction. The extreme example is shown in Figure 4B. In summary, the ECM assumption is not supported by IGS image data.

#### Belyaeva et al.

Evaluation of the enabling Dom8 identifiability assumption requires identifying inter-chromosomal triples of homologous points and computing the attendant eight distances per Figure 2B. We focus on PGP1f cells since evaluation is performed on the individual locus level (as opposed to the centroids utilized above) and the process of pronuclear fusion (via translation) and rescaling, as applied to mouse zygotes, may be too crude at this level. Homologous loci were identified after averaging 3D positions according to underlying one megabase (mb) genomic coordinate bins. To combat sparsity, we restrict attention to the top two cells (IDs 85, 26) for which >500 total reads were available for chromosomes 1–22. Even with this restriction there is a paucity of homologous loci; results only being attainable for a limited number of chromosome triples.

Formal testing of assumption of dominant *smallest* distance among eight three-way distance candidates requires operationalizing the notion of ‘dominance’. It is natural to base Dom8 evalaution on the proportion of the minimum distance to total distance: sum of all 8 distances. Formal inference surrounding this proportion will inevitably have limited power due to the exceedingly small sample size (eight distances), so we provide a more qualitative assessment.

**Figure 5.**
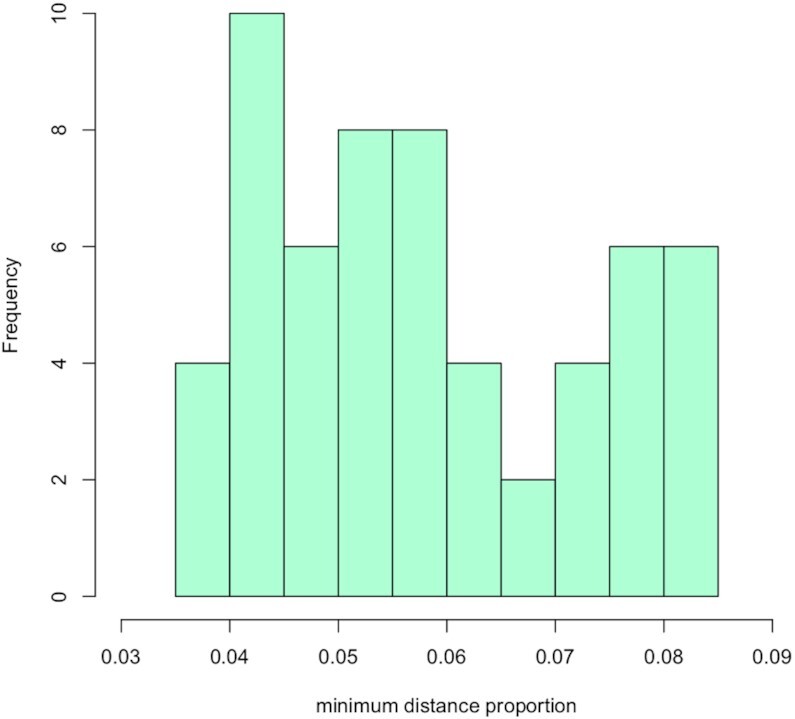
Histogram of the proportion of the minimum distance to the total distance of the eight higher-order (three-way) distances between 58 homologous loci triplets on three differing chromosomes (from the set {1, 2, 3, 4, 5} for PGP1f cell 85.

Under uniformity (all eight distances equal) the proportion of the minimum (or any) distance to the sum of the distances is 1/8 = 0.125. If we stipulate that for the minimum distance to be dominant it should be <0.125/4 we see from the histogram in Figure 5 that none of the 58 homologous triplet comparisons for cell 85 achieve this. Moreover, while cell 26 only yielded eight comparisons the minimum of these was 0.1, also notably non-dominant.

### Salvaging haploid 3D reconstruction methods

Central to our proposed rescue of existing phase-blind or implicitly haploid 3D reconstruction techniques are the phase-aware distance relationships given by (5) and (6). Again, in view of sparsity concerns, we limit formal evaluation of these assumptions to mouse zygotes, restricting analyses to (i) all chromosomes with >500 reads, or (ii) the chromosome (1 through 20) with the maximal number of reads among the 24 zygotes. Further, we focus on formal testing of (6)—equality of maternal and paternal intra-chromosomal distance matrices—since attendant data is not subject to potential artifacts associated with computational fusion of pronuclei.

**Figure 6.**
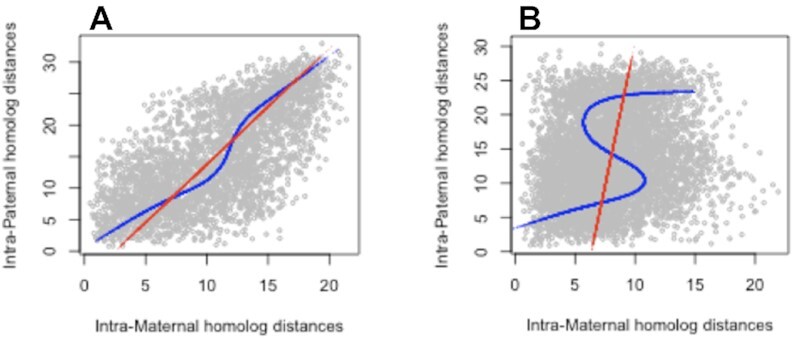
Comparison of maternal and paternal intra-homolog distances for mouse zygote 7: (**A**) chromosome 6; (**B**) chromosome 1. The red line corresponds to the first principal component while the blue line is the principal curve.

In almost all instances—25 / 27 chromosomes across seven zygotes – maternal and paternal distance matrices were significantly different (Bonferroni corrected p-value <0.01) according to GRV testing. Figure 6 illustrates these contrasting intra-homolog distances for zygote 7: panel A presents an instance (chromosome 6) of similar distances while panel B provides a representative example (chromosome 1) of distinct distances. Relationships are highlighted by superposition of principal components and principal curves (43).

**Figure 7.**
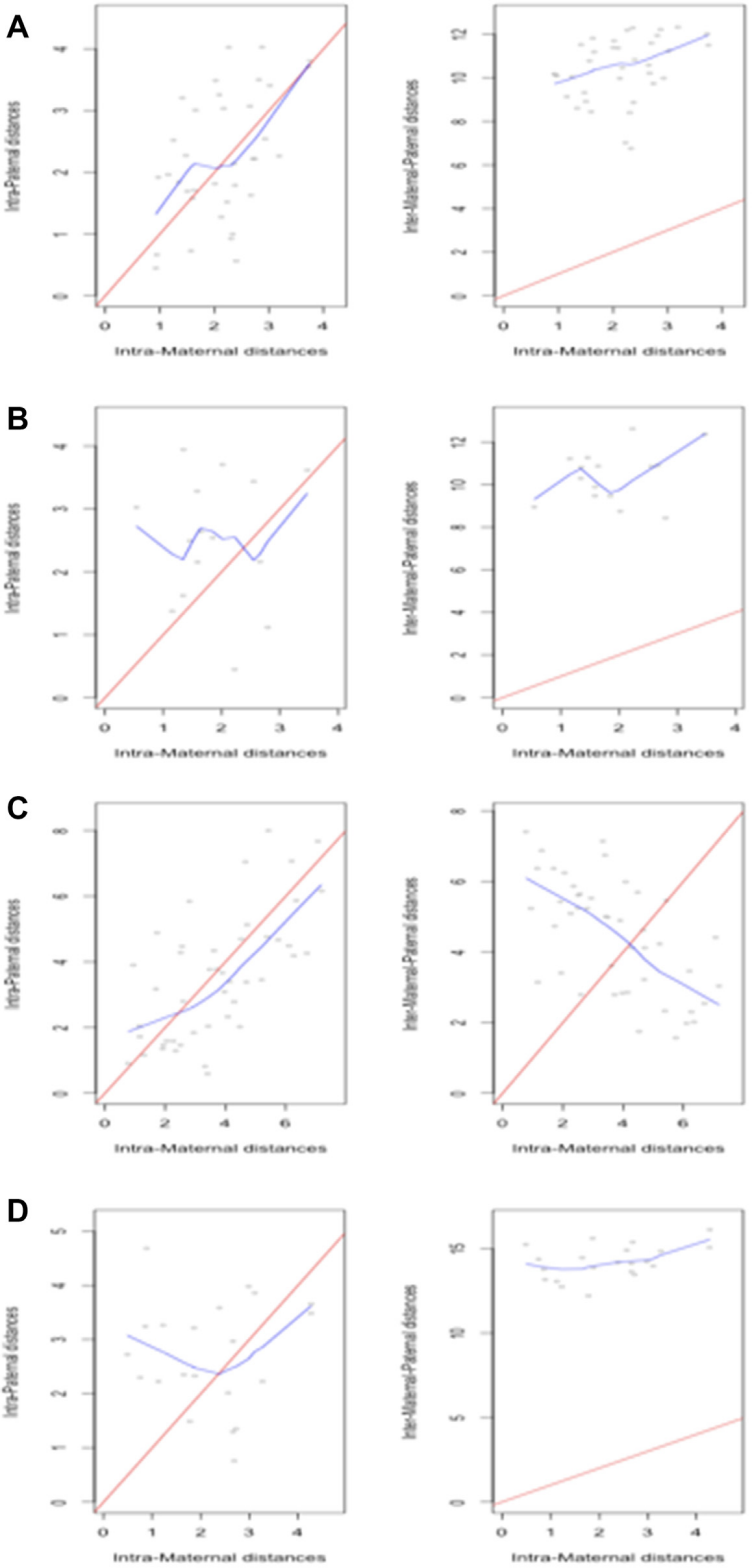
Comparison of maternal and paternal intra- and inter- homolog distances for chromosome 1 of select PGP1f cells exhibiting differing patterns with respect to salvaging assumptions: (**A**) cell 85; (**B**) cell 94; (**C**) cell 26; (**D**) cell 53. The red line corresponds to the diagonal (equality) while the blue line is a lowess smooth.

To the extent that data adequacy supports exploration in PGP1f cells, graphical checks of (5) generally affirm that inter-chromosomal distances greatly exceed their corresponding intra-chromosomal counterparts. Select results for chromosome 1 (under a relaxed 10mb binning scheme) are shown in Figure 7. The rightmost graphic in each panel displays relationships between intra-maternal and inter-maternal-paternal distances. While panel C shows comparable intra- and inter- homolog distances this is the exception over other cells and chromosomes. The leftmost graphic plots intra-maternal and intra-paternal distances, revealing instances of similarity (A, C) and absence of association (B, D), again reflective of broader findings.

Despite this preponderance of results rejecting the assumption of similar maternal and paternal distance matrices, it remains possible that the observed maternal and paternal 3D structures (as opposed to reconstructions) are similar, disagreement arising due to operating characteristics of the GRV test and the (reconstruction) process of inferring 3D structure from distance matrices. Accordingly, we pursued direct testing of actual IGS configurations, for the same chromosomes and zygotes as above, using Procrustes tests. Results were entirely concordant with GRV testing with only the same two chromosome, zygote combinations having similar configurations.

## DISCUSSION

The emergence of IGS imaging has facilitated our objective of assessing assumptions surrounding 3D *diploid* genome reconstructions based on Hi-C assays. Naturally, this begs the question as to why such reconstructions are necessary, given that IGS provides actual, rather than inferred, 3D configurations. There are at least two reasons supporting an ongoing role for reconstruction. First, both the resolution and extent of IGS assays is, at least currently, limited in comparison with Hi-C. Second, there are substantive volumes of historic Hi-C data, in a wide range of organisms and conditions, that can benefit from the added value conferred by 3D reconstruction. However, this ongoing relevance of Hi-C reinforces the need to further develop allele-specific reconstruction methods, particularly in light of the issues we identified with current approaches and our proposed salvaging scheme.

While our focus here has been on using IGS image data solely to evaluate assumptions underlying these approaches, it is possible to integrate IGS-derived features into Hi-C based reconstructions in a variety of ways. Most obvious would be the amending of constraints and assumptions when, as here, IGS finds these deficient. More sophisticated approaches involve expanding earlier analogs of integrating fluorescence *in situ* hybridization (FISH) and Hi-C data (30,44).

We have emphasized apparent violations of some of the assumptions enabling the three extant diploid 3D reconstruction techniques. In so doing we have acknowledged the limitations surrounding utilizing available IGS data for this purpose; notably, the prefusion state of maternal and paternal pronuclei in the mouse zygotes and read sparsity for PGP1f cells. However, beyond the inferred assumption violations, there are additional concerns and/or possibilities regarding these methods, which we briefly overview.

Even preceding the diploid 3D reconstruction step, the approach of (16) attempts some ambitious modeling of *inter*-chromosomal relationships between Hi-C contact counts and genomic (coordinate) distances. Their starting presumption is that a power-law relationship for *intra*-chromosomal contacts with index -1, the fractal globule model (2) can be extended genome-wide. But, the prescribed intra-chromosomal model is far from general, there being many instances of power-law violations and/or indices ≠–1 (8,13,28,29). Moreover, as demonstrated by (15), the index is (mathematically) dependent on resolution; with further dependence on organism and cell type among other factors (45). Accordingly, the notion that the joint inter-chromosomal contact : genomic distance probability distribution can be characterized by a single parameter, independent of the identities of interacting chromosomes, especially given preferential territory occupancy, seems overly simplistic.

A concern impacting the approach of (17) (which also applies to (16), pertains to initialization of allele specific configurations in the absence of any phased Hi-C data. Specifically, the manner in which *C*_*M*_, *C*_*P*_ and, especially, the corresponding *X*_*i*_, *X*_*j*_ in (2) are declared at the outset seems likely to be highly influential on resultant solutions.

As shown, the Dom8 assumption of (18) that facilitates resolving allelic ambiguity and thereby enabling 3D diploid reconstruction does not enjoy empiric support based on IGS data. This assumption followed from the definition of multi-way distances, as depicted in Figure 2A. But, alternatively, multi-way distances could be defined as the point-set diameter, which may better reflect the SPRITE cross-linking process (36). Importantly, such a definition is compatible with the Gram formulation used to effect the embedding that yields the 3D solution. A further possibility would be to utilize *tensor distances*, defined via tensor inner products and norms that generalize their vector analogs, then use embedding as implemented in multi-linear multidimensional scaling (46) to effect 3D reconstruction. Finally, distance-free reconstruction techniques may offer a natural approach to accommodating multi-way contacts.

The rigorous development of (18), establishing identifiability for diploid 3D reconstruction, is pursued in a joint, whole genome context, and the Dom8 assumption applies to multi-way inter-chromosomal contacts. However, from a data usage perspective, arguments have been made for obtaining whole genome 3D reconstructions by a staged approach, whereby single chromosome solutions are relatively positioned using (sparser) inter-chromosomal contacts (27,28). Adapting the diploid 3D reconstruction approach, and attendant assumptions, to this single allelic pair strategy might inherit the corresponding benefits.

Given the demonstrated difficulties confronting 3D diploid reconstruction algorithms based on unphased Hi-C data there is a clear upside to advancing methods for phasing Hi-C data, thereby overcoming these obstacles. While current approaches, such as HiCHap (21), achieve excellent accuracy, they are reliant on the availability of phased genomes. In turn, obtaining phased genomes often makes recourse to Hi-C data in conjunction with long-range sequencing (47). Developing pipelines that integrate these data types and algorithms, ultimately yielding phased whole genome, 3D chromatin configuration reconstructions, represents an important future objective.

## DATA AVAILABILITY

All IGS data used are available from https://www.science.org/doi/10.1126/science.aay3446 (Supplementary Tables S1 and S2).
